# Funding by Lottery: Political Problems and Research Opportunities

**DOI:** 10.1128/mBio.01369-16

**Published:** 2016-08-30

**Authors:** Adrian G. Barnett

**Affiliations:** Institute of Health and Biomedical Innovation and School of Public Health and Social Work, Queensland University of Technology, Kelvin Grove, Queensland, Australia

## LETTER

Fang and Casadevall present some excellent arguments for using a modified lottery to fund medical research (1). Research on research funding is ironically thin on the ground, but what research there is has identified significant biases and huge inefficiencies in current funding systems. If the current predominant model of funding through peer review (based on lengthy written application forms assessed by a small number of reviewers) was assessed by a grant review panel, it would likely be torn to shreds (2).

Despite the evidence of the benefits of a funding lottery, there has been only one funding agency bold enough to use it, the Health Research Council of New Zealand. The lack of uptake is likely because a lottery is unpalatable to agency staff and politicians. Warwick Anderson, the previous CEO of the Australian National Health and Medical Research Council (the largest funder of health and medical research in Australia), derisively dismissed lotteries in a speech to the National Press Club of Australia (https://npc.org.au/speakers/professor-warwick-anderson-am/).

Some critics have been suggesting that peer review is just too much hard work and perhaps a lottery would be better. Mind you this is a suggestion from economists, so take that any way you want.

We have spoken with Australian funding agencies about using a lottery, and the reaction was strongly negative, with one staff member saying, “It would make it look like we don’t know what we’re doing.” A key concern is that politicians and the public would react negatively, as a lottery might be interpreted as a lack of will to do a thorough assessment, whereas the truth is that multiple scientists around the world have tried and failed to accurately rank funding proposals; continuing to try is now unproductive and unscientific. An important barrier to using lotteries is therefore a communication issue, and we need to work with politicians, the public, and skeptical scientists to demonstrate how lotteries are fairer and less expensive than current funding systems.

Funding lotteries create an incredible opportunity to answer a tremendously important research question: “What is the impact of funding on a researcher’s career?” Previous attempts to estimate the impact of funding have used observational study designs and are therefore vulnerable to confounding, as winning funding is dependent on other characteristics, such as the scientist’s age and institution. A funding lottery creates a perfect randomized trial because we have equally worthy researchers who are funded at random. We can then track their careers from the point of randomization and compare them in terms of metrics such as publications, citations, and other funding, as well as perhaps more-complex outcomes, such as innovation. We are currently following researchers who applied for funding with the New Zealand Health Research Council and were randomly allocated funding (3); however, the sample size is small, and it may be at least a decade before we have accumulated enough data to show meaningful differences. Funding agencies considering using a lottery should also consider that it will give them the perfect data to study the impact of their funding.
